# Annotations

**Published:** 1894-04-28

**Authors:** 


### Annotations.

### Addenbrooke's Hospital, Cambridge.

Hospital committees have a tendency to work in special grooves. One hospital, for instance, is famous for lavish expenditure, another for excessive economy. At Addenbrooke's economy has been carried dangerously near to inefficiency, and has produced anomalies which we hope will disappear. It is amusing, therefore, to notice that one or two nurses with friends in Cambridge have started a cry of extravagance against the management of Addenbrooke's Hospital. The Cambridge papers have quoted a mass of figures to prove conclusively that the charges are absurdly false. Those figures show further that the hospital committee err in not voting sufficient money to adequately remunerate the staff. This is a grave error on their part, and they should at once appoint a small committee to ascertain the rate of remuneration paid at other hospitals with a view to an immediate readjustment. Nowhere do probationers have to work harder than they work at Addenbrooke's, and yet they are all of them required to pay—and to pay handsomely—to the hospital. Those members of the committee who are men of business will realise that low wages do not pay in the long run, and that no one who expects the best work will ever pay less than the current rates to his employés. The local press have taken up a very sound attitude in regard to the unfounded charges which have been made against the management of Addenbrooke's, which is probably as efficiently officered as any hospital in the country. It is an ill bird which fouls its own nest; and there can be no question that any committee which removes from their employ those who bring unfounded charges against its management are not only justified, but wise in their day and generation. Addenbrooke's Hospital is an honour to Cambridge, and we shall hope to hear that the committee have taken steps, not only to defend themselves and their officials, but to provide that henceforward the salaries and wages paid shall be more in accordance with current rates than they are at present.

### A Village Doctor: New Style.

The old-fashioned village doctor was nothing if not infallible. The less he knew the more infallible he was. If in addition to his ignorance he happened to be minus the possession of a legal qualification, his infallibity reached a maximum. Compare this with the tone of the village doctor of the *fin de siècle*. Thus speaks a modern Æsculapius to the Local Board of Haydock, a Lancashire village, in his annual report as M. O. H.: "With the permission of your board some remarks may be here offered on the mode of causation and means of prevention of typhoid fever. In so doing I would wish it to be understood that my own individual opinions and experience count for very little. As your medical officer of health it is my function to explain and interpret some little of the vast stores of knowledge which have been gradually accumulated by the collective labours of the medical profession." When a man and a doctor writes in this fashion, one unconsciously finds oneself reading on to find what more of modesty and originality there may be in his report. That report is exactly what one would expect—full, brief, scientific, and to the point. The annual report of the medical officer of health for Haydock is a model of what such reports should be. When we turn to the Medical Directory to find out who this village medical officer of health may be, we discover that he is Dr. T. E. Hayward, a St. Bartholomew's man, and that he has gained the double distinction of M.B. London and F.R.C.S. England. Such is a village doctor of to-day. There are many like him; and we congratulate the country and the medical profession on such proofs as these of the spread of science, and of the striking advance of modern medicine to influence and honour.

### Public Schools: Facts for Parents and the Press.

A North of England physician writes to us in the following terms: "I wish you would send a commissioner round to the public schools to expose their backwardness and get public inspection adopted. A man took me to X—— to see the house his boys were going to, and I was shocked at the inadequacy of the accommodation for the fees charged, *i.e.*, £150 per annum. Bathing and closet accommodation insufficient. . . . . cubic space of studies about five-hundred feet for *two* boys. The hospital has too few beds relatively to the size of the school, so that in an epidemic it gets overcrowded and then a house dormitory is turned into a ward. The bath-rooms in it are mere holes in corners, and the baths not up to time, tin and small. . . . From what I hear of Y—— it is worse than X——, and if a parent complains the reply is 'Remove the boys, we have more applications than vacancies.' . . . One of my patients, whose temperature on a Thursday was 104 degrees, was sent home on the following Tuesday. His bill for a term of eight weeks was £52 3s., and he had been buying teas and breakfasts because, *e.g.*, the fish was not fresh at those the school provided." Our correspondent furnishes us with the names of these two schools. We call them for the moment X—— and Y——. The names, however, will be published should occasion require. For the present we merely say that they are two of the largest and most famous of all our public schools. The sanitarian hardly knows what to write in such circumstances as these. A feeling of pure astonishment that such ignorance of sanitary necessities, and such wilfulness of temper, should be possible in the learned doctors of divinity who rule our public schools, is the predominant sentiment of the mind. Discipline is the prime idea inculcated upon the boys. But where is the discipline in a doctor of divinity's own mind when he wilfully shuts his eyes to the commands of elementary sanitation, commands enforced by the sternest of all penalties—the penalties of disease and death? In truth, the logic of the situation, as well as its science and religion, puts such head masters as these into a pillory of pure ridiculousness. It says to them, "Notwithstanding your early culture, notwithstanding your university distinctions, notwithstanding your position as a humble disciple, and a learned dignitary, of a great Church and religion, which have the reasonableness to care for the body as well as the soul, you are entirely ignorant of the mere elements of practical science, and, what is a thousand time worse, you are absolutely unwilling to learn." Parents and the press will surely make an end of such childish cerebration as this!

